# Supplementary material for: Quantum wake dynamics in Heisenberg antiferromagnetic chains
Source: Nat Commun. 2022 Oct 2;13:5796. doi: 10.1038/s41467-022-33571-8 (PMC9527252; doi:10.1038/s41467-022-33571-8)
Supplement: Supplementary file 1 — Supplementary Information [file 41467_2022_33571_MOESM1_ESM.pdf]

Supplemental Information for "Quantum wake dynamics in Heisenberg antiferromagnetic chains"

A. Scheie,[1,*] Pontus Laurell,[2,3] B. Lake,[4,5] S. E. Nagler,[1,6] M. B. Stone,[1] J-S Caux,[7] and D. A. Tennant[1,6,8]

[1] *Neutron Scattering Division, Oak Ridge National Laboratory, Oak Ridge, Tennessee 37831, USA*
[2] *Computational Sciences and Engineering Division, Oak Ridge National Laboratory, Oak Ridge, TN 37831, USA*
[3] *Department of Physics and Astronomy, University of Tennessee, Knoxville, TN 37996, USA.*
[4] *Helmholtz-Zentrum Berlin für Materialien und Energie GmbH, Hahn-Meitner Platz 1, D-14109 Berlin, Germany*
[5] *Institut für Festkörperphysik, Technische Universität Berlin, Hardenbergstraße 36, D-10623 Berlin, Germany*
[6] *Quantum Science Center, Oak Ridge National Laboratory, Tennessee 37831, USA*
[7] *Institute for Theoretical Physics, University of Amsterdam, PO Box 94485, 1090 GL Amsterdam, The Netherlands*
[8] *Shull Wollan Center - A Joint Institute for Neutron Sciences, Oak Ridge National Laboratory, TN 37831. USA*

(Dated: September 2, 2022)

SUPPLEMENTARY NOTE 1: REAL AND IMAGINARY $G(r,t)$

As noted in the main text, the real and imaginary parts of $G(r,t)$ probe different quantum mechanical functions. Comparing main text Eq. 2 and main text Eq. 3, one can see why the imaginary part of $G(r,t)$ goes to zero at infinite temperature or in the classical limit: as all states are equally populated, the commutator (and thus dissipations) vanish. This corresponds to $\mathcal{S}(-q,-\omega) = \mathcal{S}(q,\omega)$. Meanwhile, so long as correlations exist, main text Eq. 3 is nonzero even at infinite temperature or in the classical limit.

A nonzero commutator between spins has a non-trivial relationship to quantum entanglement. Generically, the equal time spin operators of any two different spins always commute: $[S_i^\alpha(0), S_j^\beta(0)] = 0$, no matter whether the wavefunction formed by the two spins has off-diagonal density matrix components (i.e., no matter whether the two spins are entangled). To obtain a nonzero commutator (and thus an uncertainty relation), one must introduce time evolution to one of the spins with a Hamiltonian that involves interaction between S_i and S_j. In this case, the commutator may be nonzero.

The presence of Heisenberg uncertainty generically implies quantum coherence between two operators, such that an observation of one quantity destroys the other's state. This is actually the opposite of quantum entanglement, where observation of one quantity determines the other's state. Thus, the presence of nonzero imaginary $G(r,t)$ does not necessarily imply quantum entanglement (defined by off-diagonal density matrix components), but instead it witnesses a quantum coherence between S_i and S_j. This is related (but not formally equivalent to) quantum discord, which is a generic measure of quantum correlations [1, 2]. Thus $Im[G(r,t)]$ is a witness of the quantum coherence of a system, which in the case of $KCuF_3$ extends to beyond 10 neighbors along the chain at 6 K. This is in accord with its highly coherent and entangled ground state. As temperature increases, the imaginary $G(r,t)$ becomes severely truncated in space, as shown in the main text Fig. 1.

* scheie@lanl.gov

SUPPLEMENTARY NOTE 2: FERROMAGNETIC SPIN CHAIN

As discussed in the main text, the $\pi/2$ stationary oscillations inside the quantum wake can be understood heuristically as spinon-antispinon interference. Here we propose an alternative (equally valid) heuristic for understanding the $\pi/2$ oscillations within the quantum wake: the effects of a spin-down operator on a down spin. If the $t=0, r=0$ spin is flipped up-to-down and the down-spin spinon propagates outward, the spin-lowering operator acting on a down spin results in zero. Meanwhile, the spin-lowering operator acting on an up-spin results in a spin flip. Thus odd (up-spin) sites correlations go to zero as the spinon light cone passes, and even sites flip.

To confirm the validity of these spinon heuristics, we also consider the isotropic $S=1/2$ ferromagnetic chain, and simulate its $T=0$ neutron spectra with DMRG [3–5] and LSWT, see Fig. 1. The DMRG calculation was performed on a chain of $L=50$ sites with open boundaries, keeping up to $m=500$ states in the calculation. $S(Q,\omega)$ was calculated using the DMRG++ [5] implementation of the Krylov-space correction vector method [6, 7], and a Lorentzian energy broadening with half-width at half-maximum (HWHM) $\eta = 0.1|J|$ to account for the finite-size system. To isolate the inelastic scattering, a Lorentzian with height $S(Q,0)$ was substracted at each Q-point.

Unlike the AFM case, excitations from the zero temperature FM ground state are spin flips of the same direction, which would mean no antiparticles are created and no destructive interference will occur. This is indeed

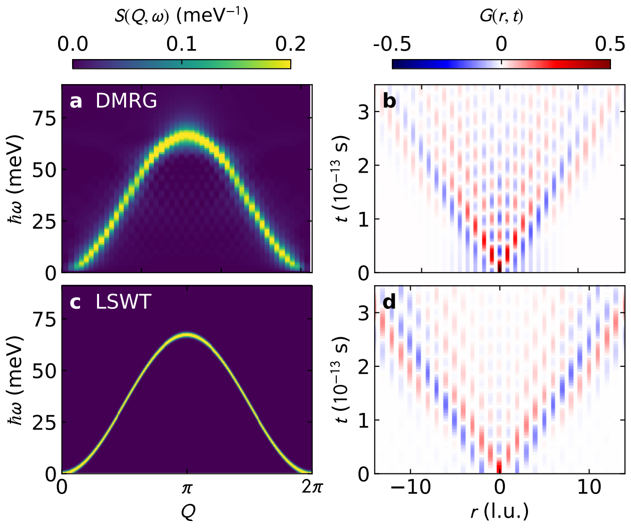

Supplementary Figure 1. Real space spin correlations for a 1D Heisenberg ferromagnetic $S = 1/2$ chain at $T = 0$, simulated with DMRG (top row) and LSWT (bottom row). Simulated neutron spectra are shown on the left (panels **a** and **c**), and Van Hove spin correlations (real part) are on the right (panels **b** and **d**). In this case, the semiclassical LSWT spin correlations are close to the DMRG quantum calculations, but the DMRG shows more oscillations near $r = 0$ at long times.

what we see: all sites oscillate in time above the light cone, and no continuum exists in $S(Q, \omega)$.

If there were regular destructive interference, it would by necessity create a continuum in the neutron spectrum $S(Q, \omega)$: well-defined oscillations in time corresponds to a sharp mode in energy, whereas suppressed (or quickly decaying) correlations correspond to diffuse modes in energy. So even without transforming the neutron data into $S(Q, \omega)$, it should be obvious from the well-defined mode that there is no significant particle-antiparticle annihilation in $G(r, t)$ for the zero temperature FM spin chain.

SUPPLEMENTARY NOTE 3: TOWARD THE ISING LIMIT

Figure 2 shows the calculated real space correlations from perturbation theory at $T = 0$ approaching the Ising limit. The $S(q, \omega)$ based off the Ising model

$$\mathcal{H} = \sum_{i=1}^{N} \left[J_{xy}(S_i^x S_{i+1}^x + S_i^y S_{i+1}^y) + J_z S_i^z S_{i+1}^z \right] \tag{1}$$

where $J_{xy} = \epsilon J_z$ and $0 < \epsilon < 1$ was calculated as described in Ref. [8] and transformed into $G(r, t)$. There are several things worth noting: first, just like the $S = 1/2$ Heisenberg chain, the odd neighbor sites' correlations go to zero, in accord with spinon-antispinon interference. Second, there is no well-defined wavefront visible in the data—possibly because the simulated intensity only includes the inelastic channel. Finally, as the Ising limit

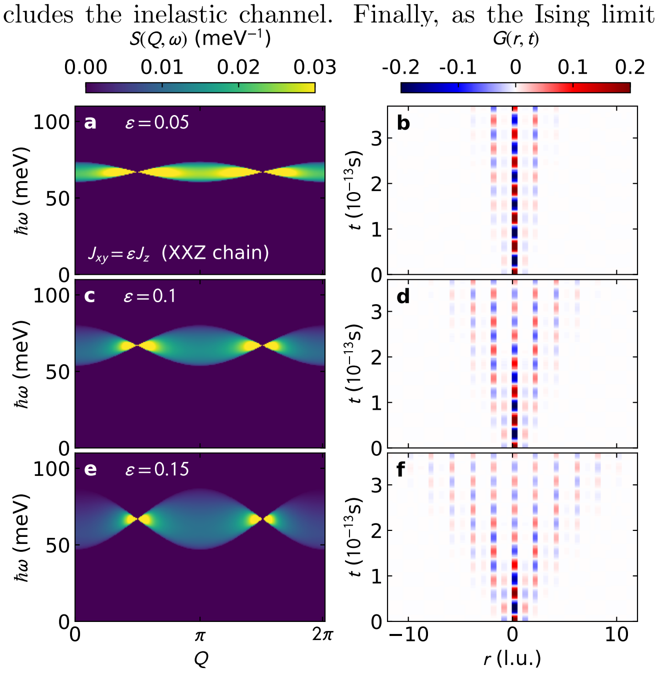

Supplementary Figure 2. Simulated spin correlations for a 1D Ising AFM chain for three different values of anisotropy. Simulated $S_{xx}(Q, \omega)$ neutron spectra are shown on the left column (panels **a**, **c**, and **e**, calculated via perturbation theory as described in Ref. [8]), and Van Hove spin correlations (real part only) are on the right column (panels **b**, **d**, and **f**). Similar to Fig. 2 in the main text, the odd neighbor sites correlations decay to zero while even neighbor sites oscillate to long times. The "light cone" gets steeper and steeper as the Ising limit is approached.

is approached, the "light cone" gets steeper and steeper, corresponding to slower and slower spinon velocities.

SUPPLEMENTARY NOTE 4: THE XY LIMIT

Although the isotropic Heisenberg chain model applicable to $KCuF_3$ can be solved exactly using the Bethe ansatz, the resulting expressions are often complicated. We can instead consider the antiferromagnetic isotropic XY-model (or XX-model) [9],

$$\mathcal{H} = J \sum_{i=1}^{N} \left[S_i^x S_{i+1}^x + S_i^y S_{i+1}^y \right], \tag{2}$$

for which simpler, closed-form expressions can be obtained using the Jordan-Wigner formalism. At zero magnetic field, for a chain of N sites with open boundary conditions, the longitudinal dynamical correlation between two lattice sites j and l can be written [10, 11]

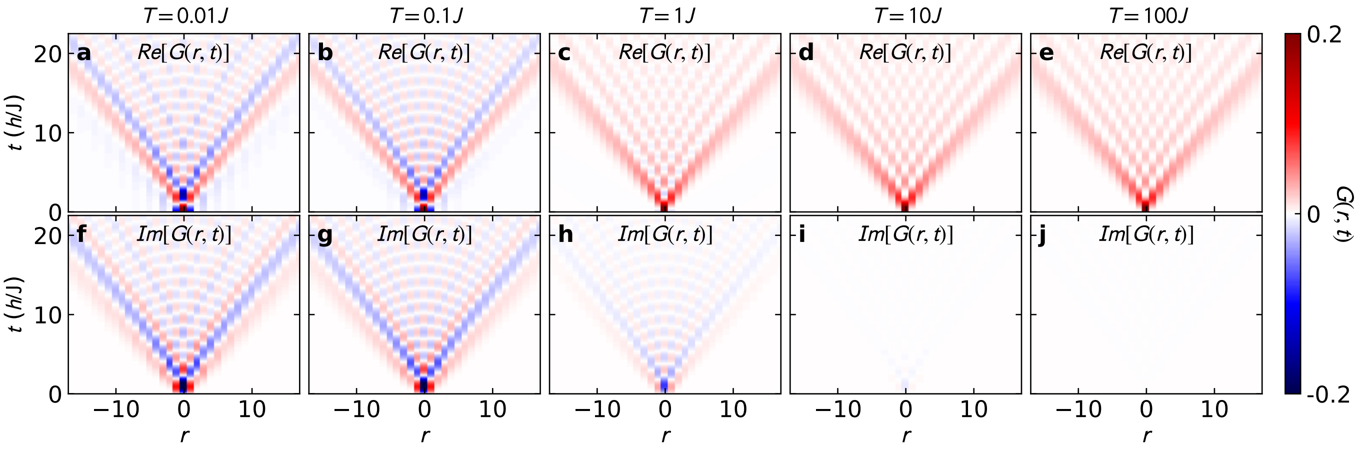

Supplementary Figure 3. Real (top row, panels **a**, **b**, **c**, **d**, and **e**) and imaginary (bottom row, panels **f**, **g**, **h**, **i**, and **j**) part of $\left\langle S_j^z(t)S_l^z(0)\right\rangle$ for the XX model, Supplementary Eq. (3), as a function of temperature. The real part becomes non-negative as T increases, whereas the imaginary part vanishes. In addition, we see a lightcone with similarly oscillatory behavior as in the Heisenberg model.

$$\begin{aligned}\left\langle S_j^z(t)S_l^z(0)\right\rangle = &\frac{1}{(N+1)^2}\left[\sum_k \left(\sin^2(kj)\right)\tanh\left(\frac{J\cos k}{2k_BT}\right)\right]\times\left[\sum_k \left(\sin^2(kl)\right)\tanh\left(\frac{J\cos k}{2k_BT}\right)\right]\\ &+\frac{1}{(N+1)^2}\left(\left[\sum_k \sin(kj)\sin(kl)\left\{\cos(tJ\cos k) - i\sin(tJ\cos k)\tanh\left(\frac{J\cos k}{2k_BT}\right)\right\}\right]^2\right)\\ &-\frac{1}{(N+1)^2}\left(\left[\sum_k \sin(kj)\sin(kl)\left\{i\sin(tJ\cos k) - \cos(tJ\cos k)\tanh\left(\frac{J\cos k}{2k_BT}\right)\right\}\right]^2\right),\end{aligned}\tag{3}$$

where $k = \frac{m\pi}{N+1}$, $1 \le m \le N+1$, is the momentum. Due to their simple structure, these sums can be evaluated at arbitrary times, temperatures and finite sizes. Yet they still capture several of the qualitative features observed in the $KCuF_3$ $G(r,t)$, as shown in Fig. 3. It is easy to analytically see the emergence of real-valued ferromagnetic correlations for all times t by considering the high-temperature limit of Supplementary Eq. (3), where the tanh factors vanish, leaving

$$\left\langle S_j^z(t)S_l^z(0)\right\rangle \approx \frac{1}{(N+1)^2}\left(\left[\sum_k \sin(kj)\sin(kl)\left\{\cos(tJ\cos k)\right\}\right]^2 + \left[\sum_k \sin(kj)\sin(kl)\left\{\sin(tJ\cos k)\right\}\right]^2\right),\tag{4}$$

which is manifestly real and non-negative for all times t. In the thermodynamic limit we have the expressions [10],

$$\left\langle S_j^z(t)S_l^z(0)\right\rangle = \begin{cases}\frac{1}{4}\left[J_{j-l}(Jt) - (-1)^l J_{j+l}(Jt)\right]^2, & \text{for } T=\infty,\\ \frac{1}{4}\left[F_{j-l}(Jt) - (-1)^l F_{j+l}(Jt)\right]^2, & \text{for } T=0,\end{cases}\tag{5}$$

where J_n is the nth Bessel function of the first kind and $F_n = J_n + iE_n$, where E_n is the Weber function.

SUPPLEMENTARY NOTE 5: TIME-LIMIT OF RELIABILITY

The long time dynamics of a Fourier transform is determined by the lowest frequencies. Consequently, the reliability of the calculated $G(r,t)$ at long times is governed by the lowest measured energy. In this case, the low-energy cutoff from the SEQUOIA experiment was 0.7 meV, which yields a cutoff in time of $\frac{h}{\hbar\omega} = 6\times10^{-12}$ s, where h is Planck's constant. However, the boundary between the MAPS and SEQUOIA data is 7 meV, which yields a slight artifact in the data and causes the $G(r,t)$

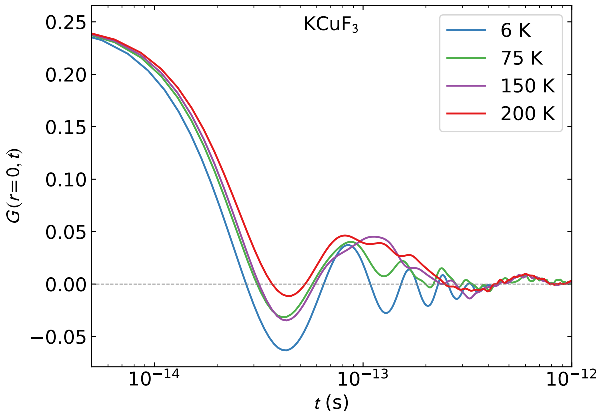

Supplementary Figure 4. On-site correlation $r = 0$ for $KCuF_3$ at various temperatures, showing the oscillations decaying. Beyond 4×10^{-13} s, the results are not reliable and the "ringing" from the low-energy cutoff begins to dominate the signal.

to "ring" with a period $\frac{h}{\hbar\omega} = 6 \times 10^{-13}$ s—this behavior is an artifact and is not physical. When the calculations are safely below this threshold, the $G(r, t)$ is reliable, as shown in Fig. 4. Although the lower energy SEQ data produced this ringing, we found that it was necessary to include in order to get a clean Fourier transform signal at higher temperatures.

To be completely safe from ringing effects, we find that one needs to stay below half the cutoff time (3×10^{-13} s for this experiment). Thus, any experiments aiming to measure $G(r, t)$ to long times must measure to appropriately high resolution and low energies.

SUPPLEMENTARY NOTE 6: QUANTUM SCRAMBLING AND OUT OF TIME CORRELATORS

Quantum scrambling is typically studied using out of time order correlators (OTOC) [12–17], which in spin chains are defined as

$$F(t) = \langle \hat{S}_a^\dagger(t) \hat{S}_b^\dagger \hat{S}_a(t) \hat{S}_b \rangle \tag{6}$$

where $\hat{S}_a(t)$ and $\hat{S}_b$ are two different spin operators at time t and $t = 0$ respectively. The OTOC is related to the commutator between these operators $Re[F(t)] = 1 - \langle |[\hat{S}_a(t), \hat{S}_b]|^2 \rangle / 2$, which functionally makes the OTOC a measure of how $\hat{S}_a(t)$ and $\hat{S}_b$ fail to commute [14]. In 1D spin chains, OTOCs reveal quantum scrambling above the light cone [16, 18, 19]. This is similar (but not identical) to imaginary $G(r, t)$, which also measures $[\hat{S}_a(t), \hat{S}_b]$ (main text Eq. 3), and thus provides similar information.

As shown in Fig. 1 and Fig. 2 of the main text, imaginary $G(r, t)$ correlations are only nonzero above the light cone, in good agreement to the commuting spin operators in the Heisenberg antiferromagnetic ground state. Above the light cone, both Bethe ansatz and $KCuF_3$ show nonzero negative static $\mathrm{Im}[G(r, t)]$ on the odd sites, and oscillating but average positive $\mathrm{Im}[G(r, t)]$ on the even sites. This concurs with quantum scrambling, where time-like separated spin operators do not commute with the spin operators at $t = 0$.

SUPPLEMENTARY REFERENCES

[1] Harold Ollivier and Wojciech H. Zurek, "Quantum discord: A measure of the quantumness of correlations," Phys. Rev. Lett. **88**, 017901 (2001).

[2] Shunlong Luo, "Quantum discord for two-qubit systems," Phys. Rev. A **77**, 042303 (2008).

[3] Steven R. White, "Density matrix formulation for quantum renormalization groups," Phys. Rev. Lett. **69**, 2863–2866 (1992).

[4] Steven R. White, "Density-matrix algorithms for quantum renormalization groups," Phys. Rev. B **48**, 10345–10356 (1993).

[5] G. Alvarez, "The density matrix renormalization group for strongly correlated electron systems: A generic implementation," Comp. Phys. Comms. **180**, 1572–1578 (2009).

[6] Till D. Kühner and Steven R. White, "Dynamical correlation functions using the density matrix renormalization group," Phys. Rev. B **60**, 335–343 (1999).

[7] A. Nocera and G. Alvarez, "Spectral functions with the density matrix renormalization group: Krylov-space approach for correction vectors," Phys. Rev. E **94**, 053308 (2016).

[8] Norikazu Ishimura and Hiroyuki Shiba, "Dynamical Correlation Functions of One-Dimensional Anisotropic Heisenberg Model with Spin 1/2. I: Ising-Like Antiferromagnets," Progress of Theoretical Physics **63**, 743–758 (1980).

[9] Elliott Lieb, Theodore Schultz, and Daniel Mattis, "Two soluble models of an antiferromagnetic chain," Ann. Phys. (N.Y.) **16**, 407 – 466 (1961).

[10] L.L. Gonçalves and H.B. Cruz, "Dynamics of the one-dimensional isotropic xy-model," Journal of Magnetism and Magnetic Materials **15-18**, 1067–1069 (1980).

[11] H B Cruz and L L Gonçalves, "Time-dependent correlations of the one-dimensional isotropic XY model," Journal of Physics C: Solid State Physics **14**, 2785–2791 (1981).

[12] Juan Maldacena, Stephen H Shenker, and Douglas Stanford, "A bound on chaos," Journal of High Energy Physics **2016**, 1–17 (2016).

[13] Brian Swingle, "Unscrambling the physics of out-of-time-order correlators," Nature Physics **14**, 988–990 (2018).

[14] Martin Gärttner, Justin G. Bohnet, Arghavan Safavi-Naini, Michael L. Wall, John J. Bollinger, and Ana Maria Rey, "Measuring out-of-time-order correlations and multiple quantum spectra in a trapped-ion quantum magnet," Nature Physics **13**, 781–786 (2017).

[15] Jun Li, Ruihua Fan, Hengyan Wang, Bingtian Ye, Bei Zeng, Hui Zhai, Xinhua Peng, and Jiangfeng Du, "Measuring out-of-time-order correlators on a nuclear magnetic resonance quantum simulator," Phys. Rev. X **7**, 031011 (2017).

[16] David J. Luitz and Yevgeny Bar Lev, "Information prop-

agation in isolated quantum systems," Phys. Rev. B **96**, 020406 (2017).

[17] Luis Colmenarez and David J. Luitz, "Lieb-robinson bounds and out-of-time order correlators in a long-range spin chain," Phys. Rev. Research **2**, 043047 (2020).

[18] Shunsuke Nakamura, Eiki Iyoda, Tetsuo Deguchi, and Takahiro Sagawa, "Universal scrambling in gapless quantum spin chains," Phys. Rev. B **99**, 224305 (2019).

[19] Hyungwon Kim and David A. Huse, "Ballistic spreading of entanglement in a diffusive nonintegrable system," Phys. Rev. Lett. **111**, 127205 (2013).
